# Pathology of Milk Sickness; an Inaugural Thesis, Presented to the Faculty of Rush Medical College, 1856

**Published:** 1857-01

**Authors:** W. H. Philips

**Affiliations:** Kenton, Ohio


THE NORTH-WESTERN

MEDICAL AND SURGICAL JOURNAL.

(NEW SERIES.)

VOL. VI. JANUARY, 1857. NO. 1.

### ORIGINAL COMMUNICATIONS.

### ARTICLE I.—*Pathology of Milk Sickness; an Inaugural Thesis, presented to the Faculty of Rush Medical College,* 1856. By W. H. PHILIPS, Kenton, Ohio.

I propose, in this paper, to consider briefly the Nature and Pathology of Milk Sickness.

In the selection of this subject, I am not unmindful of the fact, that this disease is one upon which the science of medicine has not even conferred a name, and the very existence of which is ignored by many of the Profession.

This incredulity, on the part of the Profession, is not surprising when we review the discrepant accounts of the cause, and the many dubious and speculative opinions of the nature and pathology of this disease.

That milk sickness is a disease, *sui generis*, characterized by distinct pathognomonic symptoms, we have a collection of facts too numerous and an array of testimony too respectable to admit of longer doubt. The fact that this disease has not been satisfactorily understood or explained, affords no just grounds for denying its existence, but should rather excite a spirit of inquiry and investigation that it may no longer be regarded, and so justly too, the *approbia medicorum* of this Western country.

A correct definition and classification of the available facts in any given case, is the true basis of scientific investigation; but in the present imperfect state of our knowledge of the disease under consideration, the use of the popular epithet suggestive of its cause and character will perhaps better serve our present purpose and the cause of truth than any theoretical cognomen in accordance with medical nomenclature. The direct cause of this disease is now generally conceded to be, either the ingestion of diseased animal food, or the use of the milk of animals that contains certain noxious qualities. That such is its true cause will appear from a consideration of the following facts:—

*First*—That it is invariably found to occur in the same districts, and simultaneously with a disease known as the 'Trembles' in the Graminivera.

*Second*—That a disease, corresponding in all its characteristics, does occur and can be produced at pleasure in carniverous animals, by feeding them upon the milk or flesh of animals known to have the trembles.

The question of its primary origin, is one which has an important bearing upon the pathology of this disease in the human species.

By some writers this disease has been divided into acute and chronic. But as this difference is one which is dependent rather upon the degree of development than upon any difference in the nature of the affection, we think its phases were better described as the latent, and active or acute stages of development, since it frequently subsides without ever having arrived at what is known as the active or acute form of the disease.

*Symptoms*—The first symptoms that are usually noticed, are feelings of lassitude, fatigue, stiffness of the joints after exercise, and often extreme muscular debility. This state of feebleness and want of tone in the muscular system may continue for some days or even weeks, and possibly pass off without any more serious manifestation of disease. But the more ordinary course of the disease, is, that the patient is attacked with an unusual degree of prostration; nausea and vomiting ensue; the temperature of the body is reduced, but without the ordinary sensation or appearance of chill; the patient now complains of a burning sensation in the epigastrium, with an insatiable desire for cool drinks, though the tongue is loose, flabby, and generally covered with a creamy-looking coat; the breath becomes exceedingly offensive, the peculiar fetor of which is as characteristic of this disease, as is the mercurial fetor of salivation; the face and lips become suffused, and the whole surface of the body, though cold, instead of being pale and shriveled, is quite as full as in health, and of a leaden hue, indicating an engorged state of the capillary and cutaneous vessels. The retching and vomiting still continue, the patient becomes exceedingly dull and stupid, as if laboring under the nauseating influence of tobacco or some narcotic poison. On examination, we find the respiration slow and peculiar; the pulse full, slow, soft, and compressible; the heart and large arteries throb violently, and the bowels obstinately costive.

These symptoms, the most distressing of which are the constant efforts at emesis, may continue for two or three days; and, if not relieved by the timely administration of the proper remedies, the patient becomes more dull, the epigastrium tender, the powers of locomotion are entirely arrested; all voluntary control of the muscular system is either lost or so diminished that the patient cannot be made even to protrude his tongue for examination, or if, by an extraordinary effort, he is made to do so, he has not the energy or ability to retract it, but lets it settle back by a kind of involuntary contraction; the contractions of the heart have now become very slow and feeble; the pulsations in the extremities imperceptible; the patient becomes comatose, and at length the vital energies are entirely overwhelmed and death supervenes.

Such are the ordinary symptoms and course of fatal cases of this disease. Though it may be, and frequently is, complicated with the autumnal fevers, there are usually present enough of its characteristic symptoms to make it of easy diagnosis.

*Pathology*—Up to the present time, the *materies morbi* in question has not been identified by any investigations that have been instituted; it, therefore, remains for us to arrive at a knowledge of its properties by noticing its physiological effects upon the human system. That this poisonous principle is primarily of vegetable origin, which, being ingested by the graminivera, is taken up by the lacteals and excreted with the milk, is at least a very probable proposition, and one in support of which many strong arguments can be adduced. A careful observation of the phenomena to which it gives rise, has led us to the conviction that its properties are those of a vegetable alkaloid, and not those of an animal poison, as is the received opinion. Whilst it is characteristic of the animal poisons generally to produce a dissolution of the blood and disorganization of the tissues, we have in this disease what seems to us a very different pathological condition.

It is a well-known fact, that poisons act very differently upon the human constitution, and that upon the peculiar and specific character of each depends not only its general but even its local effects — the malignant pustule producing speedy dissolution of the blood and tissues generally, whilst the poison of variola affects chiefly the dermoid structure. So with the vegetable poisons, whilst opium and its salts affect the cerebral functions, and strychnia the muscular, we see that the veratrum viride acts almost specifically upon the circulatory system, between the effects of which and the agent in question a striking analogy will be found to exist. This deleterious agent, when taken into the stomach, doubtless enters directly into the circulation, and by this means is made to bathe the extreme nervous fibrilla of every organ and tissue of the body, the primary physiological effect of which is, a diminution of the vital properties of the organic nervous system. Being thus rendered incapable of receiving and responding to the impressions of its natural stimulus, the blood, the chemico-vital changes in all of the organs are greatly diminished, and in some of them entirely suspended. Of these functional derangements, those of secretion and excretion are the most manifest.

By carefully observing the pathological conditions as they occur, we find, *First*, a diminished impetus in the systemic circulation; a congestion of the capillary vessels; deficient oxydation and de-carbonization of the blood, with diminished temperature, and an almost entire suspension of the mucous, cutaneous, biliary, and renal secretions. Though the metamorphoses of tissues are retarded, they of course continue while life lasts. Under this condition of things, therefore, we must of necessity have an accumulation of effete and excrementitious matter in the system, such as carbonic acid, biliary matter, urea, and other products of disintegration, which will of themselves, if not speedily removed, tend rapidly to overwhelm the already prostrate vital energies.

Having thus seen how it is that the organs are deprived of every necessary condition for the performance of their normal functions, we are prepared by this reasoning, *a posteriori*, to arrive at a knowledge of the cause as well as the true nature of the whole category of symptoms which characterize this disease. The points we would make, then, are:—

*First*—That this deleterious agent is possessed of a peculiar sedative quality, by which it diminishes the inherent tone and susceptibility of the organic nervous system in such a way as to impair the tonic-resisting energy of the whole capillary system, thereby inducing that *ensemble* of morbid phenomena above enumerated.

*Secondly*—That the lassitude, diminished temperature, nausea, vomiting, costiveness, fetid breath, suspended secretions, loss of muscular power, stupor, coma, and death, result not so much from any specific influence of the poison itself, as from the pathological conditions primarily induced.

In the treatment of this disease, there are two prominent indications:—*First*, to relieve the urgent symptoms; and, *Secondly*, to depurate the system of all offending matter. In the fulfilment of the first indication, which is to relieve the retching and vomiting, which is so constant and distressing, recourse may be had to the use of contra-irritation upon the epigastrium, and along the course of the spinal column. For this purpose the use of sinapisms, alternated with hot turpentine lotions, are probably the most efficient; they will usually be sufficient to procure temporary relief, which is all that can be hoped for, until the fulfilment of the second indication. This will be most readily accomplished by restoring the functions of the alimentary canal. For this purpose we would use a powder composed as follows:—

℞—Hyd. Chlor. Mite, . . . gr.ij.

Morph. Sul. . . . . . . gr.$\frac{1}{4}$.

Pul. Camph. . . . . . gr.ij.

Of these we would direct one to be taken every hour, to be followed by the use of small lumps of ice, to be swallowed after each powder. This treatment to be persevered in for twelve or even fifteen hours, after which we would direct the free exhibition of the common Seidlitz powders, to be accompanied by the use of warm stimulating enemata, and fomentations to the bowels. By this means a free evacuation of the bowels can usually be procured in from twenty-four to thirty-six hours. Should the accomplishment of this object be attempted more speedily by the use of more cogent and drastic cathartics, as is the practice of many, it is not likely to be successful; or, if so, it is almost invariably followed by the development of acute or sub-acute gastro-enteritis.

Having procured a free evacuation of the bowels, the functions of the skin and kidneys may be promoted by the use of the spts. nitri, or spts. nitri dul. with an equal quantity of the tinc. opii. camph. These being established, we would favor the elimination of excretable materials by the use of the chlorate of potassa, and such other means as favor excretion and secretion. After which, much benefit will be derived by the exhibition of small doses of strychnia, in combination with some of the vegetable tonics, for the purpose of increasing the muscular energy of the system and thereby favor a speedy convalescence.

I have thus presented a few thoughts, drawn from actual observation at the bedside—and though it may be but a dim vision of the true light, my highest ambition in this investigation has been to arrive at the knowledge of the truth.

